# Effect of Tumor Location on Clinicopathological and Molecular Markers in Colorectal Cancer in Eastern China Patients: An Analysis of 2,356 Cases

**DOI:** 10.3389/fgene.2020.00096

**Published:** 2020-02-25

**Authors:** Yaolin Song, Lili Wang, Wenwen Ran, Guangqi Li, Yujing Xiao, Xiaonan Wang, Li Zhang, Xiaoming Xing

**Affiliations:** Department of Pathology, The Affiliated Hospital of Qingdao University, Qingdao, China

**Keywords:** colorectal cancer, tumor location, clinicopathological character, RAS, BRAF, microsatellite instability

## Abstract

Colorectal cancer (CRC) has become a major health concern in China due to its increasing incidence and mortality. This study aimed to clarify the relationship between tumor locations and the clinicopathological molecular marker features in eastern China CRC patients. We continuously collected data on 2,356 CRC patients who underwent surgical resection from January 2017 to April 2019. Right-sided colorectal cancer (RCC), was located from the cecum to the transverse colon and left-side colorectal cancer (LCRC) was located from the splenic flexure to the rectum. The clinicopathological indices (including age, sex, pTNM stage, mucinous production, and distant metastasis) and frequency of molecular markers such as KRAS, NRAS, BRAF, and microsatellite instability (MSI) were statistically analyzed between the RCC and LCRC groups. The associations between clinicopathological characters and molecular markers were also investigated. LCRC and RCC proportions in eastern China CRC patients were 81.75% and 18.25%, respectively. RCC (vs. LCRC) was more frequently observed with higher frequencies of MSI-high (MSI-H) and BRAF mutations in female and younger patients, and was closely associated with metastasis, poor differentiation, and mucinous tumors. Tumor location also showed significant differences in bowel wall infiltration degree and pTNM stage. Mutation rates of KRAS, NRAS, MSI, and BRAF were 40.15%, 3.85%, 6.31%, and 2.30%, respectively. Patients with a KRAS mutation tended to be female, had mucinous, perineural invasive, and polypoid tumor. Those with NRAS mutation tended to develop well-differentiated ulcerative tumors. The BRAF mutation was more relevant with lymph node involvement, deeper infiltration of the bowel wall, mucinous, poorly-differentiated tumor with thrombus, and perineural invasion. Furthermore, MSI-H was more commonly found in younger patients with deeper bowel wall infiltration and a poorly-differentiated polypoid tumor, whereas MSS patients tended to develop lymph node involvement, and a mucinous and perineural invasive tumor. In our study, we found that LCRC and RCC showed different features on the clinicopathological and molecular markers in eastern China CRC patients. Since our data differ from those of Western countries and other regions in China, further studies are required to clarify the regional differences of the clinicopathological and molecular markers in CRC patients.

## Introduction

Colorectal cancer (CRC) is the third most common cancer, with the fourth most common cancer-related mortality worldwide (Zhang et al., 2017). In China, the incidence, mortality, and burden of CRC are all increasing due to the transition to a westernized lifestyle with obesity and physical inactivity (Center et al., 2009; Gu et al., 2018). Among Chinese patients, CRC shows the fifth highest cancer incidence in men, the fourth in women; and is fifth in cancer-related deaths in men (8.0%) and third in women (9.8%) (Feng et al., 2019).

Both genetic and environmental factors are involved in the tumorigenesis of CRC. For the past decades, studies have demonstrated that genetic molecular markers such as microsatellite instability (MSI), 18q loss of heterozygosity, and CpG island methylator phenotype (CIMP), RAS, and BRAF, among others, are closely associated with the tumorigenesis and prognosis of CRC (Sanz-Garcia et al., 2017; Saeed et al., 2019).

MSI is a genetic hypermutability condition resulting from deficient DNA mismatch repair. It is involved in various types of cancers, including colon cancer, ovarian cancer, skin cancer, and gastric cancer, among others. MSI is most frequently associated with the development of CRC and is found in about 15% of CRC tumors. RAS belongs to the proto-oncogene family that encodes three small GTPase proteins including KRAS, NRAS, and HRAS. RAS gene mutation presents in about 30% of all human cancers, and the mutation proportions of KRAS, NRAS, and HRAS are around 85%, 11-15%, and 1%, respectively (Cox et al., 2014; Chang et al., 2016). KRAS functions downstream in the epidermal growth factor receptor (EGFR) signaling pathway and is involved in cell proliferation, mutation, and cell death. KRAS mutation plays an important role in carcinogenesis, about 30-50% CRC is known to have KRAS gene mutation and the mutation positions are most frequently in codons 12 and 13, in exon 1 (Wagner et al., 2019). NRAS is closely related with KRAS; and its mutation in CRC, which is mainly located at codons 12, 13, or 61 is approximately 1-6% (Vaughn et al., 2011). BRAF is a serine kinase, downstream of KRAS, and in the MAPK signaling pathway. The most frequent mutation point of BRAF is V600E. The incidence of the BRAF V600E mutation is estimated to be about 8-10% in CRC patients (Bahrami et al., 2018; Myte et al., 2019).

CRC is anatomically divided into the right (RCC) and left CRC (LCRC) according to the location of the tumor by the border of the splenic flexure. Past studies have observed the relationship of tumor location with the development, clinicopathological characteristics, treatment, and prognosis of CRC. However, the effect of tumor location on the molecular markers of CRC still remains unclear. Recently, several studies have reported that the clinicopathological and molecular biomarkers of CRC were divergent in different regions (Giraldez et al., 2012; Perea et al., 2015). It is of great value to clarify the correlation between tumor location and genetic markers for patients in different regions. Today, most reports that study differences in molecular markers in RCC and LCRC hail from Western countries, with very few studies from eastern China.

In this study we collected data on 2,356 CRC cases from the affiliated hospital of Qingdao University, a medical center that mainly provides medical care to eastern China patients. We summarized the clinical features and molecular characteristics using RCC and LCRC. Our study provides valuable guidance and reference for the diagnosis and treatment of CRC patients in eastern China.

## Materials and Methods

### Patients

**Figure 1:**
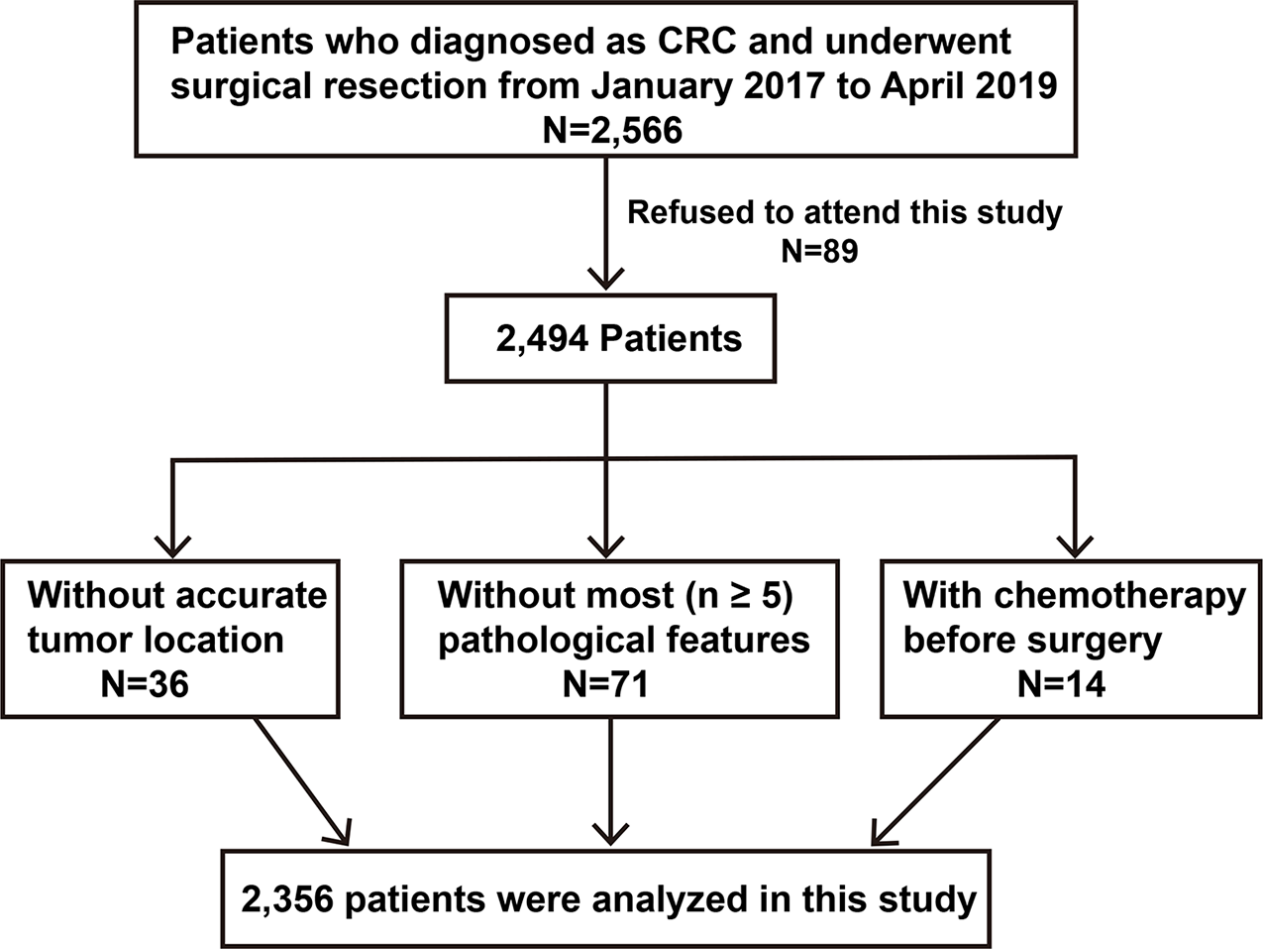
Diagram for the selection of CRC patients for this study. CRC, colorectal cancer.

We continuously collected data on 2,566 CRC patients who underwent surgical resection at the Affiliated Hospital of Qingdao University from January 2017 to April 2019. RCC was defined as CRC from the cecum to the transverse colon, while LCRC was located from the splenic flexure to the rectum. As summarized in **Figure 1**, patients who were treated with radiotherapy or chemotherapy before surgery and who lacked accurate tumor location were excluded. Finally, 2,356 cases were selected for this study analysis. For patients who developed two or more colorectal tumors, the more advanced one was selected for this study. Informed consent was obtained from all patients, and the Ethics Committee of Qingdao University approved this study.

### Genomic DNA Extraction

Fresh CRC and corresponding normal tissues were fixed by 4% paraformaldehyde at 4°C overnight, paraffin embedded, and sectioned at 5 μm for hematoxylin and eosin (HE) staining for later use. The HE section was observed under the microscope; areas that were rich in tumor cells (the proportion of tumor cells was over 20%) were selected, while non-tumor areas and necrotic areas were avoided as much as possible. Tumor tissues were scraped in a clean Eppendorf tube according to the HE section, and the genomic DNA in paraffin tissues was extracted using a Tiangen paraffin embedded tissue DNA extraction kit (Tiangen Biotech, Beijing, China), according to the manufacturer’s instructions.

### Microsatellite Instability

Microsatellite status was defined by six microsatellite markers (NR21, NR24, NR27, BAT25, BAT26, and MONO-27) and detected by an MSI detection kit (Microread, Beijing, China). Polymerase chain reaction (PCR) amplification was performed as follows: a total volume of 10 µl reaction mixture contained 20 ng of genomic DNA, 1 X PCR buffer, 1 X MSI Primer Mix, and Taq DNA Polymerase I. The running protocol was set up as follows: 5 min at 95°C once; 30 s at 94°C, 1 min at 60°C, and 1 min at 70°C for 30 cycles; 30 min at 60°C and forever at 15°C. The PCR production was diluted 1:6 and mixed with 9 µl ROX500 and HI-DI; after denaturation for 3 min at 96°C, the reaction complex was analyzed by DNA fragmentation assays (Applied Biosystems 3500DX, Massachusetts, USA). Allelic sizes were evaluated by GeneMapper software ver 4.1 (Thermo Fisher, Massachusetts, USA). MSI status was classified as Microsatellite stable (MSS), MSI-low (MSI-L, 1 marker unstable) and MSI-high (MSI-H, over 2 markers unstable).

### Mismatch Repair (MMR) Protein Immunohistochemistry

Immunohistochemistry for mismatch repair (MMR) proteins was performed as previously described (Zhang et al., 2018). Briefly, tissues were paraffin embedded and sectioned at 3 μm. Immunohistochemistry staining for MLH1, PMS2, MLH2, and MSH6 (Ventana Medical Systems Inc, Tucson, AZ, USA) was accomplished on a BenchMark XT automated staining system (Ventana Medical Systems, Inc., Tucson, AZ, USA). Two pathologists evaluated the staining results and the standards for diagnosis were as follows: expressions of all proteins were considered proficient MMR, loss of expression of one or more of the MLH1, PMS2, MLH2, and MSH6 proteins indicating DNA mismatch repair.

### KRAS, NRAS, and BRAF

Patients were tested for KRAS (codons 12 and 13), NRAS (codons 12, 13, and 61), and BRAF (V600E) to detect gene mutation; detailed detection variants are shown in **Table 1**. Genomic DNA was extracted from paraffin embedded tissues using a Tiangen kit (Tiangen Biotech, Beijing, China). RAS and BRAF mutations were detected by the Human RAS and BRAF mutation detection kit with PCR fluorescence probe, according to the manufacturer’s instructions (AmoyDx, Xiamen, China). Briefly, for each gene analysis, 25 µl total volume of complex mixture containing 0.3 μm primers and Taqman probes, 200 μm dNTPs, 200 μm Taq polymerase, and 90 ng of DNA. PCR amplification were set up using ABI 7500 as follows: 42°C, 5 min; 94°C, 3 min (94°C, 15 s; 60°C, 60 s); 40 cycles. The running data were analyzed by 7500 software ver 2.3 (Applied Biosystems, Massachusetts, USA).

**Table 1:** Detection of the amino acid (AA) alternations of KRAS, NRAS, and BRAF.

Markers	Codon	Amino acid
KRAS	Codon 12	G12D
		G12A
		G12R
		G12C
		G12V
		G12S
	Codon 13	G13C
		G13D
NRAS	Codon 12	G12S
		G12C
		G12D
		G12A
		G12V
	Codon 13	G13R
		G13D
		G13V
	Codon 61	Q61K
		Q61R
		Q61L
		Q61H1
		Q61H2
BRAF	Codon 600	V600E

### Statistical Analysis

All data were analyzed by SPSS 19.0.0 statistical analysis software (IBM Corp., Armonk, NY, USA). The relationship between the two groups (RCC and LCRC) was evaluated using a standard chi-square test. Data that were not qualified for the chi-square test, were merged into the groups to reach the standard. *p* < 0.05 was defined as statistically significant.

## Results

### Clinicopathological Characteristics by RCC and LCRC

**Figure 2:**
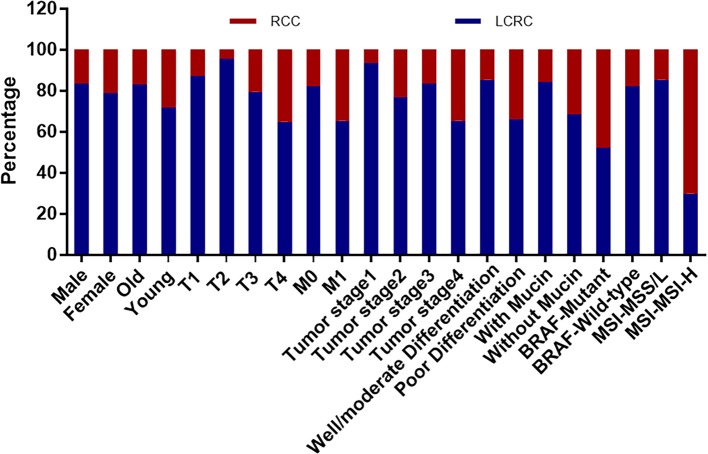
The correlation between clinicopathological characteristics and tumor locations that shows statistically significant difference. Compared with LCRC, RCC is more relevant with higher frequencies of MSI-high (MSI-H) and BRAF mutation, and higher incidence in female and younger patients, and was closely associated with bowel wall invasion, metastasis, poor differentiation, and mucinous tumors. (LCRC, left-sided colorectal cancer; RCC, right-sided colorectal cancer; MSI, microsatellite instability; MSS/L, microsatellite stable plus microsatellite instability-low; MSI-H, microsatellite instability-high; Old, ≥50 years old; Young, <50 years old; T, bowel wall invasion; M, metastasis).

The summary of the basic clinicopathological indices with respect to the tumor location is shown in **Table 2** and **Figure 2**. Of the 2,356 CRC patients, 81.75% (95% confidence interval [CI]: 79.8-83.7%) were LCRC and 18.25% (95% CI: 16.3-20.2%) were RCC. RCC was more frequently associated with younger female patients (*p* = 0.000), a higher risk of metastasis (*p* = 0.003), poorly-differentiated carcinoma (*p* = 0.000), and mucin production (*p*=0.000). Tumor location was also involved in the infiltration degree of the bowel wall and pTNM stage. In contrast, LCRC and RCC showed no significant differences in lymph node involvement, polypoid gross type, tumor, and perineural invasion.

**Table 2:** Clinicopathological characteristics and tumor location.

Pathological Characteristics	Number N = 2356	LCRC N = 1926 (81.7%)	RCC N = 430 (18.3%)	*P* value (RCC vs. LCRC)
Gender	100% N = 2356			0.000 ^a,^*
Male	60.9% N = 1437	83.6% N = 1201	16.4% N = 236	
Female	39.1% N = 919	78.9% N = 7 25	21.1% N = 194	
Age	100% N = 2356			0.000 ^a,^*
≥50 years	88.4% N = 2083	83.1% N = 1730	16.9% N = 353	
<50 years	11.6% N = 273	71.8% N = 196	28.2% N = 77	
T	100% N = 2356			0.000 ^a,^*
T1	2.0% N = 47	87.2% N = 41	12.8% N = 6	
T2	15.0% N = 354	95.8% N = 339	4.2% N = 15	
T3	80.6% N = 1898	79.5% N = 1509	20.5% N = 389	
T4	2.4% N = 57	64.9% N = 37	35.1% N = 20	
N	99.7% N = 2349			0.332 ^a^
N0	55.9% N = 1314	80.7% N = 1061	19.3% N = 253	
N1	25.5% N = 600	82.5% N = 495	17.5% N = 105	
N2	18.6% N = 435	83.7% N = 364	16.3% N = 71	
M	99.9% N = 2355			0.003 ^a,^*
M0	97.8% N = 2303	82.1% N = 1891	17.9% N = 412	
M1	2.2% N = 52	65.4% N = 34	34.6% N = 18	
Tumor stage	99.7% N = 2349			0.000 ^a,^*
1	13.3% N = 313	93.6% N = 293	6.4% N = 20	
2	41.5% N = 976	76.9% N = 751	23.1% N = 225	
3	42.9% N = 1008	83.5% N = 842	16.5% N = 166	
4	2.3% N = 52	65.4% N = 34	34.6% N = 18	
Gross type	99.9% N = 2354			0.098 ^a^
Polypoid	22.8% N = 537	79.3% N = 426	20.7% N = 111	
Ulcerative	77.2% N = 1817	82.5% N = 1499	17.5% N = 318	
Differentiation	99.9% N = 2355			0.000 ^a,^*
Well/moderate	81.3% N = 1915	85.3% N = 1634	14.6% N = 281	
Poor	18.7% N = 440	66.1% N = 291	33.9% N = 149	
Mucin production	100% N = 2356			0.000 ^a,^*
Without	83.2% N = 1960	84.4% N = 1654	15.6% N = 306	
With	16.8% N = 396	68.7% N = 272	31.3% N = 124	
Tumor thrombus	99.7% N = 2349			0.672 ^a^
Without	73.3% N = 1722	81.2% N = 1412	18.8% N = 310	
With	26.7% N = 627	81.2% N = 509	18.8% N = 118	
Perineural invasion	99.7% N = 2348			0.788 ^a^
Without	57.2% N = 1344	81.5% N = 1095	18.5% N = 249	
With	42.8% N = 1004	82.0% N = 823	18.0% N = 181	
KRAS	80.6% N = 1898			0.179 ^a^
Mutant	40.1% N = 762	80.6% N = 614	19.4% N = 148	
Wild-type	59.9% N = 1136	83.1% N = 944	16.9% N = 192	
NRAS	80.6% N = 1898			0.218 ^a^
Mutant	3.8% N = 73	87.7% N = 64	12.3% N = 9	
Wild-type	96.2% N = 1825	81.9% N = 1494	18.1% N = 331	
BRAF	42.6% N = 1003			0.001 ^b,^*
Mutant	2.3% N = 23	52.2% N = 12	47.8% N = 11	
Wild-type	97.7% N = 980	82.3% N = 807	17.7% N = 173	
MSI	98.9% N = 2329			0.000 ^a,^*
MSS/L	93.7% N = 2182	85.4% N = 1863	4.6% N = 319	
MSI-H	6.3% N = 147	29.9% N = 44	70.1% N = 103	

^a^Pearson’s chi-squared test, ^b^Yates’s correction for continuity, *Statistically significant.

LCRC, left-sided colorectal cancer; RCC, right-sided colorectal cancer; MSI, microsatellite instability; MSS/L, microsatellite stable plus microsatellite instability-low; MSI-H, microsatellite instability-high.

### Molecular Differences in RCC and LCRC

We checked the MSI, KRAS, NRAS, and BRAF mutation status in this study. For MSI detection, among the 2,329 cases, 757 cases were analyzed by immunohistochemistry staining for MMR proteins and 1,572 cases were analyzed by PCR; both DNA mismatch repair and MSI-H results were considered. Since MSI-L CRC showed no difference with the MSS tumor (Pawlik et al., 2004), we merged the MSI-L (n = 3) and MSS (n = 2179) tumor as MSS/L, which differed from MSI-H (n = 147). Among the 2,356 patients with CRC, the detection rates of molecular markers were 80.56% for KRAS and NRAS, 42.57% for BRAF, and 98.85% for MSI. The mutation rates for these molecular markers were 40.15%, 3.85%, 2.30%, and 6.31% for KRAS, NRAS, BRAF, and MSI, respectively. RCC was significantly associated with higher incidence of MSI-H (*p* = 0.000) and BRAF mutation (*p* = 0.001) compared with LCRC. However, there was no measurable difference between RCC and LCRC on the KRAS and NRAS mutation. Furthermore, we checked the correlation between the genetic markers and found that the KRAS mutation was accompanied with a lower mutation rate of NRAS, while the mutation status of KRAS and BRAF was incompatible, no association was found between the KRAS mutations and the BRAF mutation.

### Associations Between Molecular Markers and Clinicopathological Features

**Figure 3:**
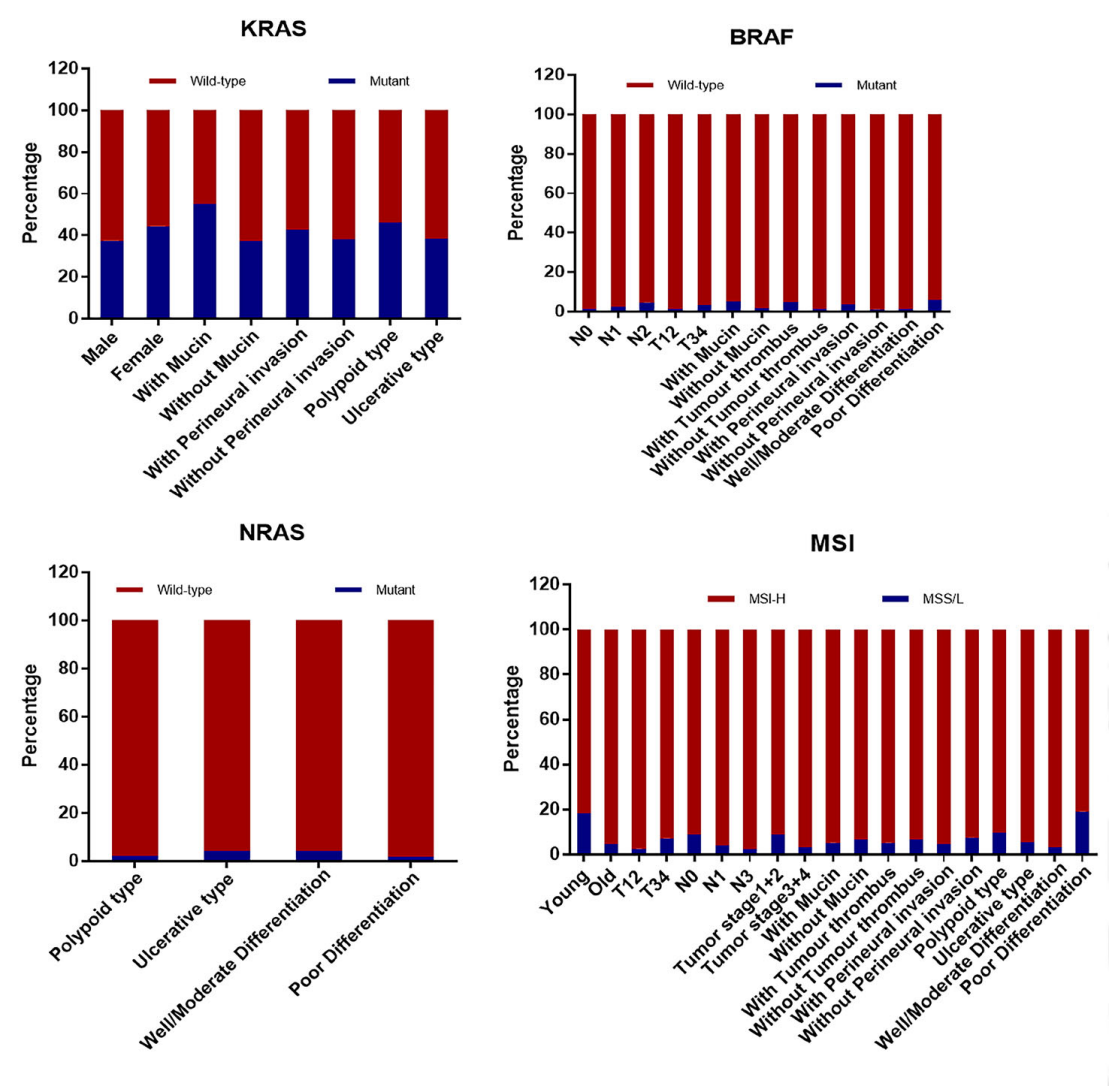
The correlation between clinicopathological characteristics and molecular markers that shows statistically significant difference. KRAS mutation more commonly occurred in female patients with a mucinous, perineural invasive, and polypoid tumor while NRAS mutation is more associated with well-differentiated ulcerative tumors. BRAF mutation was more relevant to lymph node involvement, deeper infiltration of the bowel wall, mucinous, a poorly-differentiated tumor with thrombus, and perineural invasion. MSI-H was more commonly found in younger patients with deeper bowel wall infiltration and a poorly-differentiated polypoid tumor. (MSI, microsatellite instability; MSS/L, microsatellite stable plus microsatellite instability-low; MSI-H, microsatellite instability-high; Old, ≥50 years old; Young, <50 years old; T, bowel wall invasion; N, lymph node involvement; M, metastasis).

We further analyzed the relationship between molecular markers and clinicopathological features. As **Table 3** and **Figure 3** shows, KRAS mutation more frequently occurred in female CRC patients (*p* = 0.003), with mucinous (*p* = 0.000), perineural invasive (*p* = 0.046), and polypoid tumor (*p* = 0.004). On the contrary, NRAS mutation was significantly associated with ulcerative (*p* = 0.043) and well/moderately-differentiated tumor (*p* = 0.040). BRAF mutation was more related with lymph node metastasis (*p* = 0.030), bowel wall invasion, mucin production (*p* = 0.010), tumor thrombus (*p* = 0.002), perineural invasion (*p* = 0.018), poor differentiation (*p* = 0.001) and MSI status including MSS/L and MSI-H. MSI-H status was more frequently involved in patients below the age of 50 (*p* = 0.000), had deeper bowel wall infiltration (*p* = 0.001), polypoid gross type (*p* = 0.001), and a poorly-differentiated (*p* = 0.000) tumor. Whereas, MSS/L status was more commonly associated with lymph node involvement (*p* = 0.000), mucinous (*p* = 0.000), and a perineural invasion (*p* = 0.007) tumor.

**Table 3:** Correlation between clinicopathological characteristics and molecular marker status.

Pathological Characteristics	KRAS				NRAS				BRAF				MSI			
	Number N = 1898	MUT 40.1%	WT 59.9%	*P* value	Number N = 1898	MUT 3.8%	WT 96.2%	*P* value	Number N = 1003	MUT 2.3%	WT 97.7%	*P* value	Number N = 2329	MUT 6.3%	WT 93.7%	*P* value
Gender	100% N = 1898			0.003 ^a,^*	100% N = 1898			0.222 ^a^	100% N = 1003			0.667 ^a^	100% N = 2329			0.601 ^a^
Male	60.6% N = 1150	37.4% N = 430	62.5% N = 720		60.6% N = 1150	3.4% N = 39	96.6% N = 1111		61.5% N = 617	2.1% N = 13	97.9% N = 604		60.8% N = 1417	6.1% N = 86	93.9% N = 1331	
Female	39.4% N = 748	44.4% N = 332	55.6% N = 416		39.4% N = 748	4.5% N = 34	95.5% N = 714		38.5% N = 386	2.6% N = 10	97.4% N = 376		39.2% N = 912	6.7% N = 61	93.3% N = 851	
Age	100% N = 1898			0.417 ^a^	100% N = 1898			0.057 ^a^	100% N = 1003			0.949 ^c^	100% N = 2329			0.000 ^a,^*
<50 years	11.3% N = 215	42.8% N = 92	57.2% N = 123		11.3% N = 215	1.4%N=3	98.6% N = 212		10.5% N = 105	2.9% N = 3	97.1% N = 102		11.5% N = 268	18.3% N = 49	91.7% N = 219	
≥50 Years	88.7% N = 1683	39.8% N = 670	60.2% N = 1013		88.7% N = 1683	4.2%N=70	95.8% N = 1613		89.5% N = 898	2.2% N = 20	97.8% N = 878		88.5% N = 2061	4.8% N = 98	95.2% N = 1963	
Bowel wall invasion (T)	100% N = 1898			0.813 ^a^	100% N = 1898			N/A	100% N = 1003			N/A	100% N = 2329			N/A
T1	2.1% N = 40	35% N = 14	65% N = 26		2.1% N = 40	0	40		2.1% N = 21	0	21		2% N = 47	8.5% N = 4	91.5% N = 43	
T2	15.1% N = 286	38.5% N = 110	61.5% N = 176		15.1% N = 286	2.8% N = 8	97.2% N = 278		14.0% N = 140	1.4% N = 2	98.6% N = 138		15.0% N = 348	1.7% N = 6	98.3% N = 342	
T3	80.2% N = 1523	40.6% N = 619	59.4% N = 904		80.2% N = 1523	4.1%N = 63	95.9% N = 1460		81.1% N = 813	2.2% N = 18	97.8% N = 795		80.6% N = 1877	6.9% N = 129	93.1% N = 1748	
T4	2.6% N = 49	38.8% N = 19	61.2% N = 30		2.6% N = 49	4.1%N = 2	95.9% N = 47		2.8% N = 29	10.3% N = 3	89.7% N = 26		2.4% N = 57	14.0% N = 8	86% N = 49	
T12	17.2% N = 326	38.0% N = 124	62% N = 202	0.42 ^a^	17.2% N = 326	2.5% N = 8	97.5% N = 318	0.159 ^a^	16.1% N = 161	1.2% N = 2	98.8% N = 159	0.408 ^a^	17.0% N = 395	2.5% N = 10	97.5% N = 385	0.001 ^a,^*
T34	82.8% N = 1572	638	934		82.8% N = 1572	65	1507		93.9% N = 842	2.5% N = 21	97.5% N = 821		83% N = 1934	7.1% N = 137	92.9% N = 1797	
Lymph node involvement (N)	99.7% N = 1893			0.475 ^a^	99.7% N = 1893			0.304 ^a^	99.8% N = 1001			0.030 ^a,^*	99.7% N = 2323			0.000 ^a,^*
N0	55.8% N = 1057	39.7% N = 420	60.3% N = 637		55.8% N = 1057	3.4% N = 36	96.6% N = 1021		53.2% N = 533	1.3% N = 7	98.7% N = 526		55.8% N = 1297	8.7% N = 113	91.3% N = 1184	
N1	25.2% N = 478	42.3% N = 202	57.7% N = 276		25.2% N = 478	5.0% N = 24	95% N = 454		27.1% N = 271	2.6% N = 7	97.4% N = 264		25.6% N = 594	4.0% N = 24	96% N = 570	
N2	19% N = 358	38.3% N = 137	61.7% N = 221		19% N = 358	3.6%N = 13	96.4% N = 345		19.7% N = 197	4.6% N = 9	95.4% N = 188		18.6% N = 432	2.3% N = 10	97.7% N = 422	
Distant metastasis (M)	99.9% N = 1897			0.500 ^a^	99.9% N = 1897			0.619 ^a^	100% N = 1003			1.000 ^c^	99.9% N = 2328			1.000 ^b^
M0	98.0% N = 1860	40% N = 744	60% N = 1116		98.0% N = 1860	3.8% N = 71	96.2% N = 1789		98.1% N = 984	2.3% N = 23	97.7% N = 961		97.8% N = 2276	6.3% N = 144	93.7% N = 2132	
M1	2% N = 37	45.9% N = 17	54.1% N = 20		2.0% N = 37	5.4%N = 2	94.6% N = 35		1.9% N = 19	0	19		2.2% N = 52	5.8% N = 3	94.2% N = 49	
Tumor stage	99.7% N = 1893			0.735 ^a^	99.7% N = 1893			0.536 ^a^	99.8% N = 1001			N/A	98.6% N = 2323			1.000 ^c^
I	13.5% N = 255	37.6% N = 96	62.4% N = 159		13.5% N = 255	2.4% N = 6	97.6% N = 249		12.5% N = 125	0	125		13.3% N = 310	3.2% N = 10	96.8% N = 300	
II	41.3% N = 782	40.0% N = 313	60.0% N = 469		41.3% N = 782	3.8% N = 30	96.2% N = 752		39.9% N = 399	1.8% N = 7	98.2% N = 392		41.4% N = 962	10.6% N = 102	89.4% N = 860	
III	43.3% N = 819	40.7% N = 333	59.3% N = 486		43.3% N = 819	4.3%N = 35	95.7% N = 784		45.8% N = 458	3.5% N = 16	96.5% N = 442		43.0% N = 999	3.2% N = 32	96.8% N = 967	
IV	1.9% N = 37	45.9% N = 17	54.1% N = 20		1.9% N = 37	5.4% N = 2	94.6% N = 35		1.8% N = 19	0	19		2.2% N = 52	5.8% N = 3	94.2% N = 49	
I+II	54.8% N = 1037	39.4% N = 409	60.6% N = 628	0.540 ^a^	54.8% N = 1037	3.5% N = 36	96.5% N = 1001	0.401 ^a^	52.3% N = 524	1.3% N = 7	98.7% N = 517	0.036 ^a,^*	54.8% N = 1272	8.8% N = 112	91.2% N = 1160	0.000 ^a,^*
III+IV	45.2% N = 856	40.9% N = 350	59.1% N = 506		45.2% N = 856	4.3%N = 37	95.7% N = 819		47.7% N = 477	3.4% N = 16	96.6% N = 461		45.2% N = 1051	3.3% N = 35	96.7% N = 1016	
Mucin production	100% N = 1898			0.000 ^a,^*	100% N = 1898			0.341 ^a^	100% N = 1003			0.010 ^c,^*	98.6% N = 2323			0.000 ^a,^*
with	17.0% N = 322	55.0% N = 177	45.0% N = 145		17.0% N = 322	2.8% N = 9	97.2% N = 73		17.0% N = 171	5.3% N = 9	94.7% N = 162		26.7% N = 620	5.3% N = 33	94.7% N = 587	
without	83.0% N = 1576	37.1% N = 585	62.9% N = 991		83.0% N = 1576	4.1% N = 64	95.9% N = 1512		83.0% N = 832	1.7% N = 14	98.3% N = 818		73.3% N = 1703	6.6% N = 113	93.4% N = 1590	
Tumor thrombus	99.7% N = 1892			0.425 ^a^	99.7% N = 1892			0.419 ^a^	99.7% N = 1000			0.002 ^a,^*	98.6% N = 2323			0.288 ^a^
with	26.4% N = 500	38.6% N = 193	61.4% N = 307		26.4% N = 500	3.2% N = 16	96.8% N = 484		2.7% N = 268	4.9% N = 13	95.1% N = 255		26.7% N = 620	5.3% N = 33	94.7% N = 587	
without	73.6% N = 1392	40.7% N = 567	59.3% N = 825		73.6% N = 1392	4.1% N = 57	95.9% N = 1335		97.3% N = 732	1.4% N = 10	98.6% N = 722		73.3% N = 1703	6.6% N = 113	93.4% N = 1590	
Perineural invasion	99.6% N = 1891			0.046 ^a,^*	99.6% N = 1891			0.719 ^a^	99.6% N = 999			0.018 ^a,^*	99.7% N = 2322			0.007 ^a,^*
with	45.4% N = 859	42.6% N = 366	57.4% N = 493		45.4% N = 859	4.1% N = 35	95.9% N = 824		47.9% N = 479	3.5% N = 17	96.5% N = 462		42.8% N = 994	4.7% N = 47	95.3% N = 947	
without	54.6% N = 1032	38.0% N = 393	62.0% N = 639		54.6% N = 1032	3.7% N = 38	96.3% N = 994		52.1% N = 520	1.2% N = 6	98.8% N = 514		57.2% N = 1328	7.5% N = 100	91.5% N = 1228	
Gross types	99.9% N = 1896			0.004 ^a,^*	99.9% N = 1896			0.043 ^a,^*	100% N = 1003			0.451 ^a^	99.9% N = 2327			0.001 ^a,^*
Polypoid type	22.7% N = 431	46.2% N = 199	53.8% N = 232		22.7% N = 431	2.1% N = 9	97.9% N = 422		23.0% N = 231	3.0% N = 7	97.0% N = 224		22.8% N = 531	9.6% N = 51	90.4% N = 480	
Ulcerative type	77.3% N = 1465	38.4% N = 562	61.6% N = 903		77.3% N = 1465	4.3% N = 63	95.7% N = 1402		77% N = 772	2.1% N = 16	97.9% N = 756		77.2% N = 1796	5.3% N = 96	94.7% N = 1700	
Differentiation	99.9% N = 1897			0.066 ^a^	99.9% N = 1897			0.040 ^a,^*	100% N = 1003			0.001 ^c,^*	99.9% N = 2328			0.000 ^a,^*
Well/Moderate	82.3% N = 1561	39.2% N = 612	60.8% N = 949		82.3% N = 1561	4.3% N = 67	95.7% N = 1494		81.7% N = 819	1.5% N = 12	98.5% N = 807		81.2% N = 1891	3.3% N = 63	96.7% N = 1828	
Poor	17.7% N = 336	44.6% N = 150	55.4% N = 186		17.7% N = 336	1.8% N = 6	98.2% N = 330		18.3% N = 184	6.0% N = 11	94.0% N = 173		18.8% N = 437	19.2% N = 84	80.8% N = 353	

^a^Pearson’s chi-squared test, ^b^Yates’s correction for continuity, ^c^Fisher’s exact test, N/A not available, *Statistically significant.

Mut, mutant; Wt, wild-type.

### BRAF Mutation was Specifically Low in Eastern China CRC Patients

Among the 1,003 CRC patients with BRAF test, 23 patients with a positive BRAF mutation was found with a mutation rate of 2.29% - much lower than that in the published data of 8-10% (Bahrami et al., 2018; Myte et al., 2019). This result indicated that BRAF mutation rate was specifically low in eastern China patients. Also, with respect to the tumor location, the mutation rates for LCRC and RCC were 1.49% and 6.36%, respectively. We analyzed the mutation rate of BRAF in each pTNM stage and found that it was mostly detected in stage III (n = 18/23, 78.26%).

## Discussion

RCC and LCRC were first proposed as two distinct tumors by Bufill (1990), from the perspective of molecular genetics. The clinical performance, prognosis, and sensitivity to targeted therapy differs significantly depending on the tumor location. For the past decade, the role of molecular markers in the diagnosis and prognosis of cancer has been increasingly prominent. Since the indices of cancer vary in different regions and people, clarifying the effect of colorectal tumor location on clinicopathological features and molecular markers in regional areas is of great value for clinicians.

In this study, for the eastern China patients, the frequency of RCC was 18.25%, lower than in previous reports from other regions in China such as Shanghai (24.4% and 25.3%), Shantou (36.9%), Tianjin (50.6%), and Guangdong (17.5%), as well as Japan (26.3% and 29.3%) (Xu et al., 2010; Watanabe et al., 2012; Liu et al., 2017; Peng et al., 2017; Qin et al., 2017; Natsume et al., 2018; Guo et al., 2019), whereas the RCC frequency in the United States was as high as 42% (Siegel et al., 2014). This finding indicates that the distribution of RCC in eastern China patients might be different compared to those of other regions in China and Western countries.

For the RCC and LCRC clinicopathological characteristics, our findings were similar to those in published data. RCC was more frequently associated with female patients, metastasis, mucinous, poorly-differentiated carcinoma, and a higher correspondence with the BRAF mutation and MSI-H status (Wangefjord et al., 2013; Li et al., 2015; Fu et al., 2019). Furthermore, our results showed that RCC was more common in younger patients who were under 50 years old, providing a diagnostic reference for clinicians.

For the molecular markers in CRC, the mutation rates of MSI-H, KRAS, and NRAS were reported to be 6-15% (Horvat and Stabuc, 2011; Gelsomino et al., 2016; Chang et al., 2017; Samstein and Chan, 2017), 38.5-40% (Natsume et al., 2018), and 1-6% respectively (Downward, 2003). In our study, the frequencies of the MSI-H (6.31%), KRAS (40.15%), and NRAS (3.85%) mutation were in accordance with those in the Western countries and other regions in China, whereas the BRAF (2.29%) mutation was specifically lower. The BRAF mutation rate was reported to be around 2.5%-6.15% in different regions of China (Peng et al., 2017; Wang et al., 2018), and 3.7-11.3% in other Asian countries (Lee et al., 2015; Fujiyoshi et al., 2017; Natsume et al., 2018), for the eastern China patients, this incidence was 2.23%, much lower than those of Western countries, reported as 17-19.4% (Hutchins et al., 2011; Loupakis et al., 2015). We analyzed the pTNM stage and found that the BRAF mutation was mostly detected at stage III which was in line with previous data (Sayagues et al., 2018). The low mutation rate in eastern China patients might be due to the regional difference and varying genetic predispositions. Further studies need to be completed to investigate the effect of environmental and genetic factors on the molecular markers. Furthermore, several reports showed that the frequency of the BRAF mutation in RCC was around 4-10% in Asia, and in our data at around 6.36%, in agreement with the published data. RCC more frequently corresponded with a higher mutation rate of MSI-H and BRAF but had no association with the KRAS and NRAS mutation. Also, as **Table 4** shows, we checked the mutation status of RAS and BRAF and found them to be irreconcilable, this was in accordance with previous reports (De Roock et al., 2010).

**Table 4:** The interactions between molecular marker status.

	KRAS			NRAS			BRAF		
	Mut	WT	*P* value	Mut	WT	*P* value	Mut	WT	*P* value
**NRAS**			0.000 ^a,^*			N/A			0.624 ^a^
**Mut**	2	71		N/A	N/A	N/A	0	22	
**WT**	760	1065		N/A	N/A	N/A	38	931	
**BRAF**			0.000 ^a,^*			0.624 ^a^			N/A
**Mut**	0	22		0	22		N/A	N/A	N/A
**WT**	395	574		38	931		N/A	N/A	N/A
**MSI**			0.112 ^a^			0.124 ^a^			0.163 ^a^
**MSI-H**	37	75		1	111		3	58	
**MSS/L**	721	1041		71	1691		20	911	

^a^Pearson’s chi-squared test, N/A not available, *Statistically significant.

Mut, mutant; Wt, wild-type; MSI, microsatellite instability; MSI-H, microsatellite instability-high; MSS/L, microsatellite stable plus microsatellite instability-low.

We additionally analyzed the correlation between the molecular markers and basic pathological features. The KRAS mutated tumor was reported to be more frequent in female patients with mucinous differentiation and polypoid growth (Wangefjord et al., 2013; Li et al., 2015). In our study, we found it was more frequently observed in female patients, accompanied with perineural invasion, mucin production, and polypoid gross type tumor, which is mainly consistent with the published data from Western countries. As another member of the RAS family, NRAS mutation is rare and shows no significant relevance with histologic features (Irahara et al., 2010); nevertheless, we discovered that it was associated with a well/moderate differentiation grade and ulcerative carcinoma. The BRAF-mutated tumor was more often related with pathologic characteristics such as a location on the right-side, lymph-node metastases, mucin component, tumor thrombus, perineural invasion, and low differentiation grade. These findings were in agreement with existing studies (Li et al., 2015). The MSI-H phenotype has been reported to be involved in poor differentiation, mucinous histology, and right-sided colon location (Battaglin et al., 2018). Moreover, we found that it was regularly observed in younger patients (<50 years old, *p* = 0.000), a deeper infiltration of bowel wall, and polypoid gross type tumor. The distribution of the correlations between molecular markers and histologic features in our study were not completely in accordance with the published data of Western countries and other regions in China, and we assume that this difference is a consequence of multiple sample sizes and regional diversity.

For the treatment of potentially resectable colon cancer with RAS and BRAF wild-type status, LCRC with FOLFOXIRI ± cetuximab is recommended to patients, while FOLFOXIRI ± bevacizumab is recommended for RCC patients. For patients with RAS or BRAF mutation, regardless of tumor location, FOLFOXIRI ± bevacizumab is recommended (Falcone et al., 2007).

For the palliative treatment of colon cancer, further classification has been made with respect to the tumor location. In first line treatment, patients with both KRAS, NRAS, and BRAF wild-type are suitable for intense medical treatment, doublet chemotherapy plus cetuximab is recommended to the LCRC patients and doublet chemotherapy plus bevacizumab is preferred for RCC patients (Tejpar et al., 2017). For those who are not suitable to undergo intense care but have an MSH status, immune checkpoint inhibitors are recommended. In second line treatment, despite the status of the RAS/BRAF gene, immune checkpoint inhibitors are recommended for patients with an MSH status (Diaz and Le, 2015; Overman et al., 2018). Patients who are RAS wild-type and BRAF V600E mutated are recommended a VIC regimen. In third line treatment, despite the status of the RAS and BRAF gene, fruquinitinib is recommended (Li et al., 2018; Yuan et al., 2019).

There were several limitations in our study: (1) a single medical center study; (2) lack of clinical treatment data such as applications of target drugs, chemotherapy, or radiotherapy; (3) no track of survival data; and (4) other molecular markers like CIMP, 18q loss of heterozygosity, CIMP were not included in this research.

In summary, our study collected data on 2,356 cases of CRC and analyzed the relationship between tumor location, clinicopathological, and molecular features. We found that RCC (vs LCRC) was significantly associated with a higher incidence of the MSI-H and BRAF mutation but showed no measurable difference on the KRAS and NRAS mutation. Considering the limited reports published on the correlation between tumor location and molecular markers for eastern China patients, our study provides a valuable reference for physicians and researchers to study CRC.

## Data Availability Statement

The datasets generated for this study are available on request to the corresponding author.

## Ethics Statement

Informed consent of all patients was obtained and the Ethic Committee of Qingdao University approved this study.

## Author Contributions

YS and XX conceived and designed the study. XX contributed reagents, protocols, and materials. WR, GL, YX, and XW conducted the experiments and analyzed the raw experimental data. LZ provided pathological diagnosis. YS and LW collected and statistically analyzed the data. YS wrote the paper. XX modified the manuscript.

## Funding

This work was supported with funding from the National Natural Science Foundation of China (Grant No.81201947, 81972329); the Natural Science Foundation of Shandong (Grant No. ZR2009CM014); the Excellent Young Scientist Foundation of Shandong Province (Grant No. 2006BSB14001); and the Qingdao minsheng science and technology project (Grant No. 17-3-3-38-nsh).

## Conflict of Interest

The authors declare that the research was conducted in the absence of any commercial or financial relationships that could be construed as a potential conflict of interest.

## Abbreviations

CIMP, CpG island methylator phenotype; CRC, colorectal cancer; HE, hematoxylin and eosin; LCRC, left-sided colorectal cancer; MMR, mismatch repair; MSI, microsatellite instability; MSI-H, microsatellite instability-high; MSS/L, microsatellite stable plus microsatellite instability-low; PCR, polymerase chain reaction; RCC, right-sided colorectal cancer.
